# A rare case of multisystem autoimmune disease presenting with retinopathy and alopecia post COVID-19 vaccination

**DOI:** 10.1186/s12348-026-00605-x

**Published:** 2026-06-08

**Authors:** Srinivasan Sanjay, Samanthila Waduthantri, Manju Chandran, Soon-Phaik Chee

**Affiliations:** 1 Singapore National Eye Centre, 11 Third Hospital Avenue, Singapore, 168751 Singapore; 2 Osteoporosis and Bone Metabolism Unit, Department of Endocrinology, Singapore General Hospital (SGH), Life Centre (Level 4), Outram Road, Singapore, 169608 Singapore; 3 Singapore Eye Research Institute, The Academia, 20 College Road, Discovery Tower Level 6, Singapore, 169856 Singapore; 4 Department of Ophthalmology, Yong Loo Lin School of Medicine, National University of Singapore, NUHS Tower Block, Level 7, 1E Kent Ridge Road, Singapore, 119228 Singapore

**Keywords:** Autoimmune retinopathy, Antiretinal antibodies, Immunosuppression, Multimodal imaging, COVID-19, Vaccination

## Abstract

**Purpose:**

To report a case of autoimmune retinopathy (AIR) with multiple systemic involvement

**Results:**

A 45-year-old Asian Indian male presented with progressive blurring of vision in the right eye for five years. Initial best-corrected visual acuity (BCVA) was 20/80 in RE and 20/25 in LE. Clinical evaluation was normal except for a few pigmentary alterations in the periphery. Optical coherence tomography (OCT) later revealed ellipsoid zone loss, and fundus fluorescein angiography showed peripheral venular leakage. Uveitis workup was negative except for a positive T-spot TB; Anti-tubercular therapy for nine months didn’t improve the symptoms, with BCVA declining to 20/400 in RE. Fundus autofluorescence demonstrated hypo-autofluorescence in the macula surrounded by hyper-autofluorescence. Review of history revealed dermatological features, including alopecia areata and scalp vitiligo, followed by ocular disease and systemic autoimmune abnormalities. The history also indicated onset about two months after COVID-19 vaccination. Antiretinal and other systemic antibodies were positive. The patient was diagnosed with auto immune retinopathy (AIR) and treated with mycophenolate mofetil, escalating to 1.5 g twice daily. At final follow-up 6 months later, BCVA improved to 20/200 in RE and remained stable in LE, with reduced autofluorescence abnormalities.

**Conclusion:**

We report a case of multi system autoimmune disease initially presenting with dermatological features, followed by ocular and other systemic features which may have been triggered following COVID-19 vaccination in an otherwise healthy Indian male. We propose it could be a temporal event and are unable to prove a causal factor. A combination of multimodal imaging with multi-disciplinary approach helped us to come to a final diagnosis in this arduous journey for the patient.

## Introduction

We hereby report a case of autoimmune retinopathy associated with multisystem involvement possibly following Coronavirus disease − 19 (COVID-19) vaccination in an otherwise healthy Indian male.

COVID-19 vaccination has been reported to have a temporal association with development of episcleritis, scleritis, anterior uveitis, intermediate uveitis, posterior uveitis, Vogt Koyanagi Harada like disease and optic neuritis [1]. In addition there was reported cases of viral anterior uveitis involving herpes and varicella zoster viruses [1]. There has been no report of autoimmune retinopathy and systemic antibodies following COVID-19 or it's vaccination in medical literature. Autoimmune retinopathies (AIRs) comprise a rare but clinically significant group of immune-mediated retinal disorders characterized by progressive, unexplained visual dysfunction [2]. These conditions typically present with combined central and peripheral vision loss, often accompanied by visual field defects such as ring scotomas. Electroretinography (ERG) is a key diagnostic modality, revealing generalized attenuation of rod and cone responses, indicative of photoreceptor dysfunction [2]. Circulating antiretinal autoantibodies (ARAs), which are implicated in retinal injury through mechanisms such as molecular mimicry and antigen-specific cytotoxicity, are seen. Fundoscopic findings are variable, ranging from a normal appearance to vascular attenuation, diffuse or localized retinal atrophy, pigmentary changes, and occasionally waxy pallor of the optic nerve head. Importantly, intraocular inflammation is minimal or absent, distinguishing AIR from other uveitic entities [2]. Recent guideline from the American Academy of Ophthalmology advocate a multimodal diagnostic approach, integrating clinical presentation, imaging modalities (including optical coherence tomography and fundus autofluorescence), electroretinogram (ERG) abnormalities, and serologic confirmation of ARAs to classify cases as probable, possible, or unlikely AIR [2]. Despite these advances, AIR remains diagnostically challenging due to overlapping phenotypes, variable antibody pathogenicity, and the absence of standardized therapeutic protocols. Current literature emphasizes the need for further research into the immunopathogenesis of AIR and the development of evidence-based treatment strategies [2, 3]. 

## Results

A 45-year-old Indian man presented with a 5-year history of blurring vision in the right eye (RE), worse during the day than at night. He was born to parents in a consanguineous marriage; no other family members had an eye disease. Five years earlier, another center had diagnosed him with rod–cone dystrophy.

**Fig. 1:**
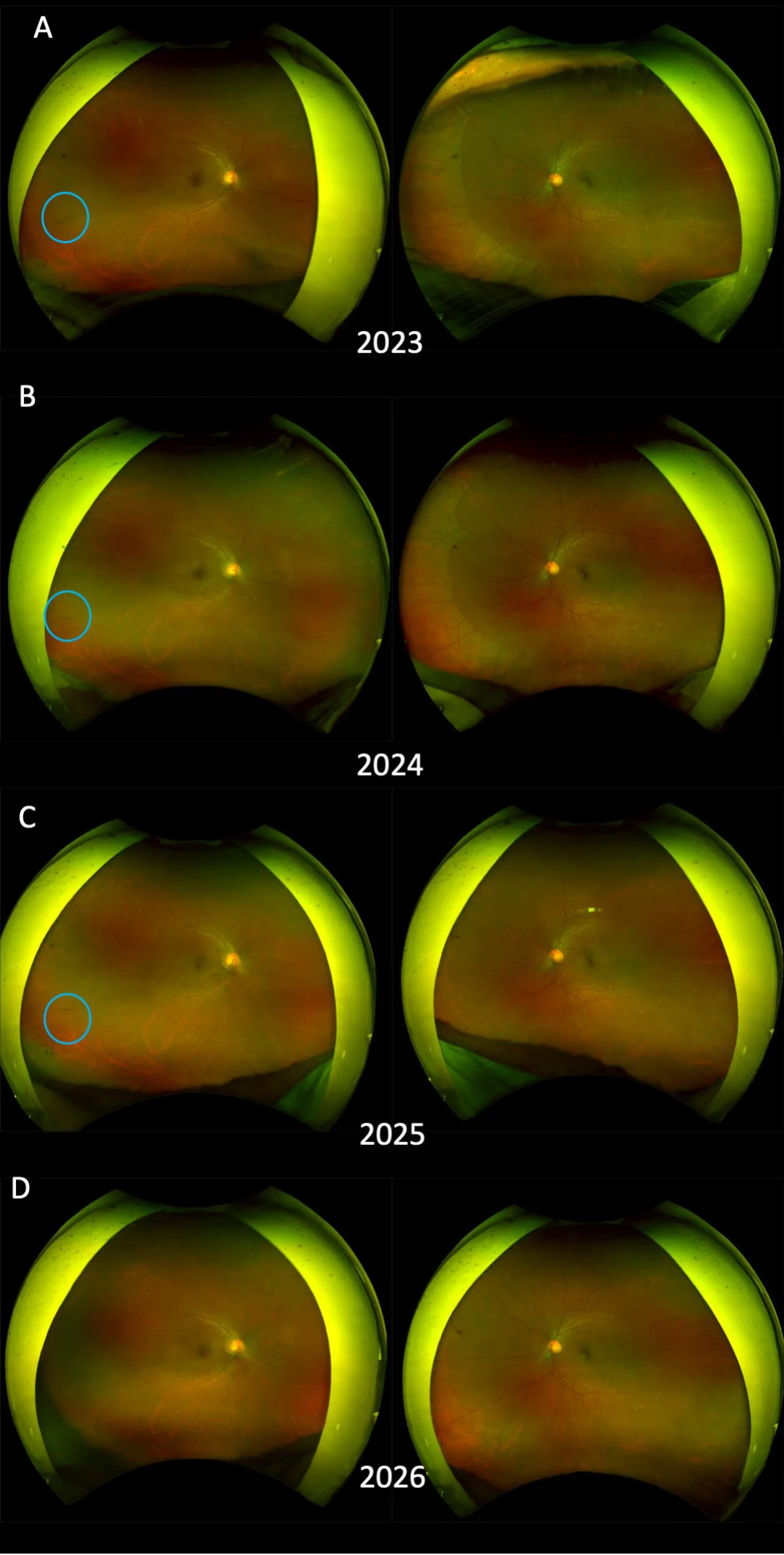
Showing ultrawide optos images in 2023–2026 with no discernible changes except peripheral pigmentation in the RE (blue circle)

**Fig. 2:**
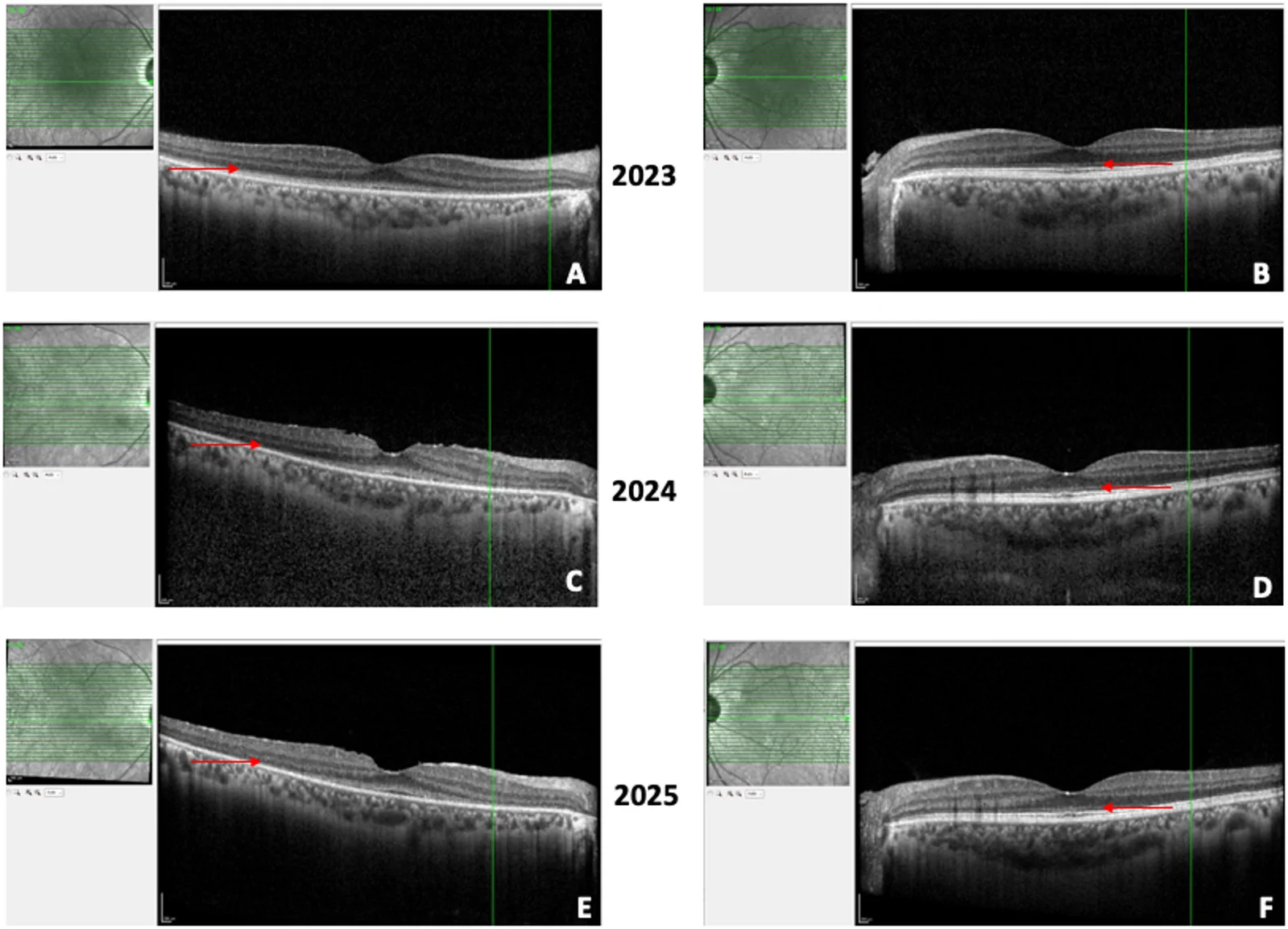
Shows optical coherence tomography changes in the RE (A/C/E) and B/D/F LE. The red arrow shows progressive profound loss of ellipsoid zone in RE and granular changes in LE and restoration of EZ in LE in 2025 sub foveally

**Fig. 3:**
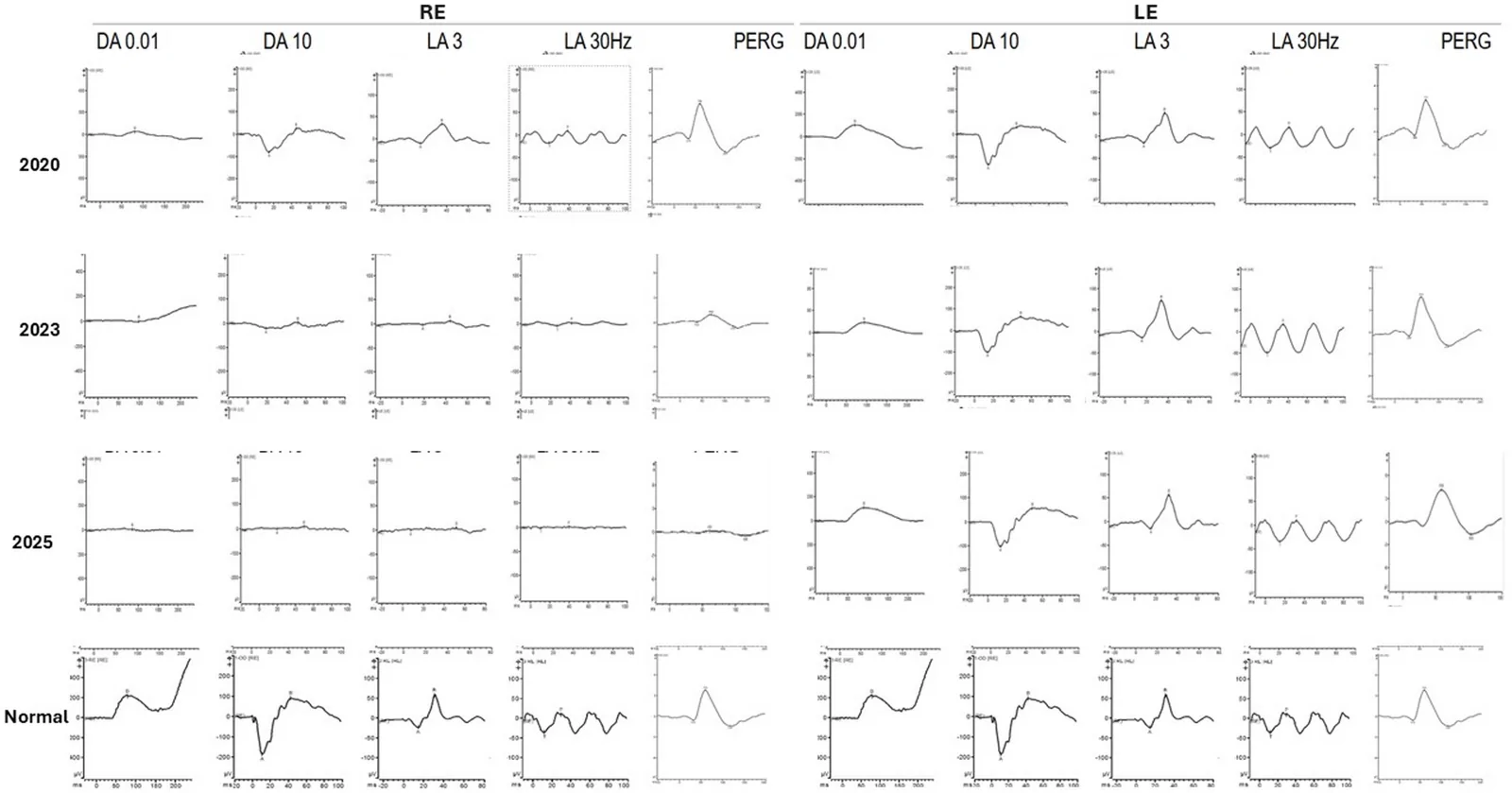
Shows the electroretinography tracings of the patient’s right eye (RE) and left eye (LE) in the years 2020, 2023, 2025. DA- Dark adaptation, LA – Light adaptation, PERG- Pattern ERG. The eyes are compared with normal tracing at the bottom. 2020-RE scotopic dim flash(rod), maximal (mixed rod-cone), as well as the photopic (cone) derived responses were significantly reduced with reduction in the amplitude. The 30 Hz flicker responses were delayed. LE was within normal limits. PERG P50 was symmetrical in both eyes indicative of a normal macular function. 2023- RE full field ERG was undetectable and decreased in amplitude, maximal response was reduced compared to LE. Photopic response was delayed and decreased. LE scotopic, maximal, photopic response was decreased but not delayed. The 30 Hz was delayed in RE and normal in LE. PERG P50 was reduced in RE indicative of macular dysfunction. and normal in LE. 2025- 6 months after initiation of immunosuppressive therapy. RE responses were severely attenuated and LE remained stable compared to 2023

At the time of that diagnosis, best-corrected visual acuity (BCVA) was 20/80 in the RE and 20/25 in the left eye (LE). The anterior segment was normal. Fundus examination of the RE showed perivascular pigmentation in the temporal periphery; the LE fundus was normal (Fig. 1A, B). Optical coherence tomography (OCT) demonstrated patchy loss of the ellipsoid zone (Fig. 2A [RE], B [LE]). Available electroretinogram (ERG) tracings showed reduced dark adaptation (DA) and light adaptation (LA), worse in the RE than the LE (Fig. 3, top row) indicating a bilateral asymmetric disease.

**Fig. 4:**
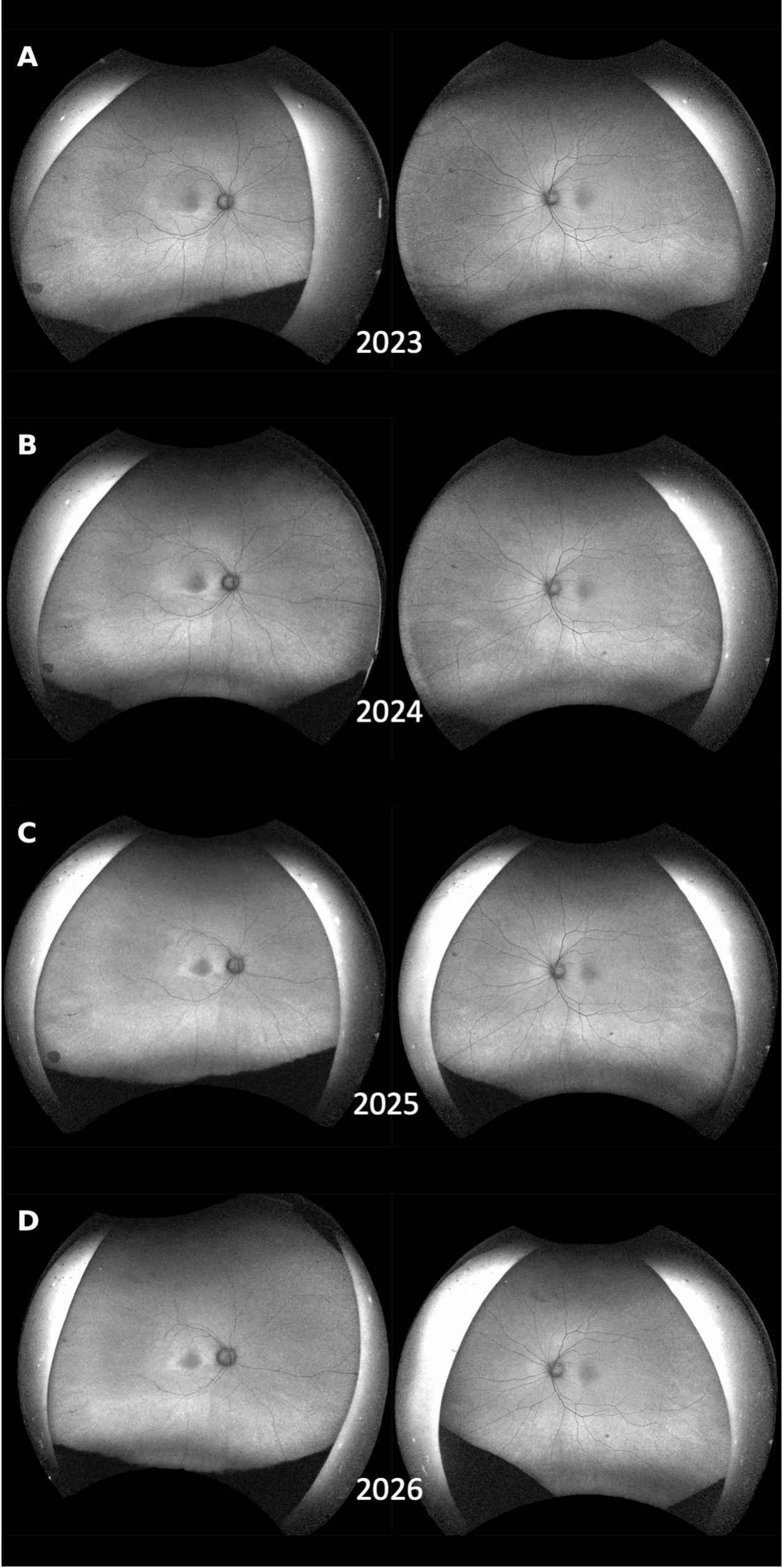
Shows Fundus autofluorescence images 2023–2026, RE and LE. RE serial images showing hypo autofluorescence in the macula area surrounded by the hyperautofluorescence, which reduced in 2024 after treatment and subsequently reduces further in 2024. LE serial images showing hyper autofluorescence in LE more prominent in peripapillary area and also in macular area which reduced following treatment

The patient sought a second opinion for progressive RE blur; clinical features were essentially unchanged. Fundus autofluorescence (AF) revealed bilateral central hypo-autofluorescence surrounded by diffuse hyper-autofluorescence in the RE and peripapillary hyper-autofluorescence (Fig. 4A, B).

Correlating images of OCT and AF in 2023,2024 (Figs. 2 and 4) we noted that there is smudging or discontinuity of EZ on OCT with hyper-autofluorescence on AF as a result of retinal pigment epithelium accumulating excess lipofuscin from shedding of outer segments.

**Fig. 5:**
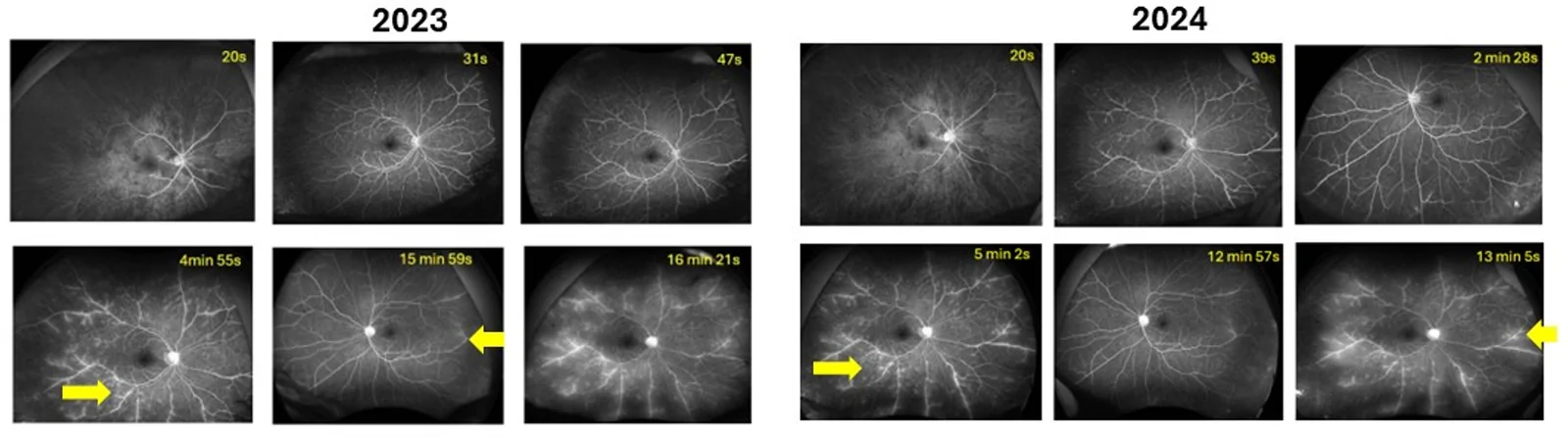
Shows Fundus fluoroscein angiography in 2023 showing normal arm- retina time with progressive venular leak (yellow arrow) in the later phases in RE. There is a mild temporal leak in the periphery in the LE. 2024- 9 months after Anti tubercular treatment and steroids, there is no change in the FFA pattern

Fundus fluorescein angiography (FFA) showed predominant retinal venous leakage in both eyes, more profuse in the RE (Fig. 5).

A detailed uveitis workup including full blood count, C-reactive protein (CRP), erythrocyte sedimentation rate (ESR), Veneral Diseases Research Laboratory (VDRL) and treponema pallidum particle agglutination (TPPA). Mantoux was non- reactive and only T-SPOT.TB was positive. Immune work up for vasculitis like antinuclear antibody (ANA), antineutrophil cytoplasmic antibodies (ANCA) and anti-double strand DNA were negative. A diagnosis of presumed Tubercular (TB) vasculitis was considered, and anti-tubercular treatment (ATT) with a four-drug regimen (isoniazid, rifampicin, pyrazinamide, ethambutol) was initiated alongside oral prednisolone 50 mg in a tapering dose, in consultation with infectious disease team. Systemic and ocular complications were monitored during 9 months of therapy. Ethambutol-related optic toxicity was monitored every 3 months through optic function assessment (pupil examination, red desaturation, and Ishihara screening), clinical optic nerve evaluation, and OCT measurements of retinal nerve fiber layer and ganglion cell-inner plexiform layer thickness.

After ATT, BCVA declined to 20/400 in the RE and was 20/125 in the LE. AF and FFA were unchanged compared with pre-ATT and after oral steroid therapy (Figs. 4C and D and 5). ERG (Fig. 3, middle row) showed near-complete flattening of both dark-adapted and light-adapted responses in the RE and reduced DA in the LE.

At our center, further testing revealed positive anti-retinal antibodies (carbonic anhydrase, aldolase, enolase, pyruvate kinase). Positron emission tomography (PET) without fluorodeoxyglucose demonstrated an incidental avid pinpoint subpleural nodule in the lateral segment of the right middle lobe, a thyroid nodule, and multiple small ametabolic liver cysts. Doppler ultrasonography of the carotid and vertebral arteries was normal. Systemic TB was ruled out by the infectious diseases team.

**Fig. 6:**
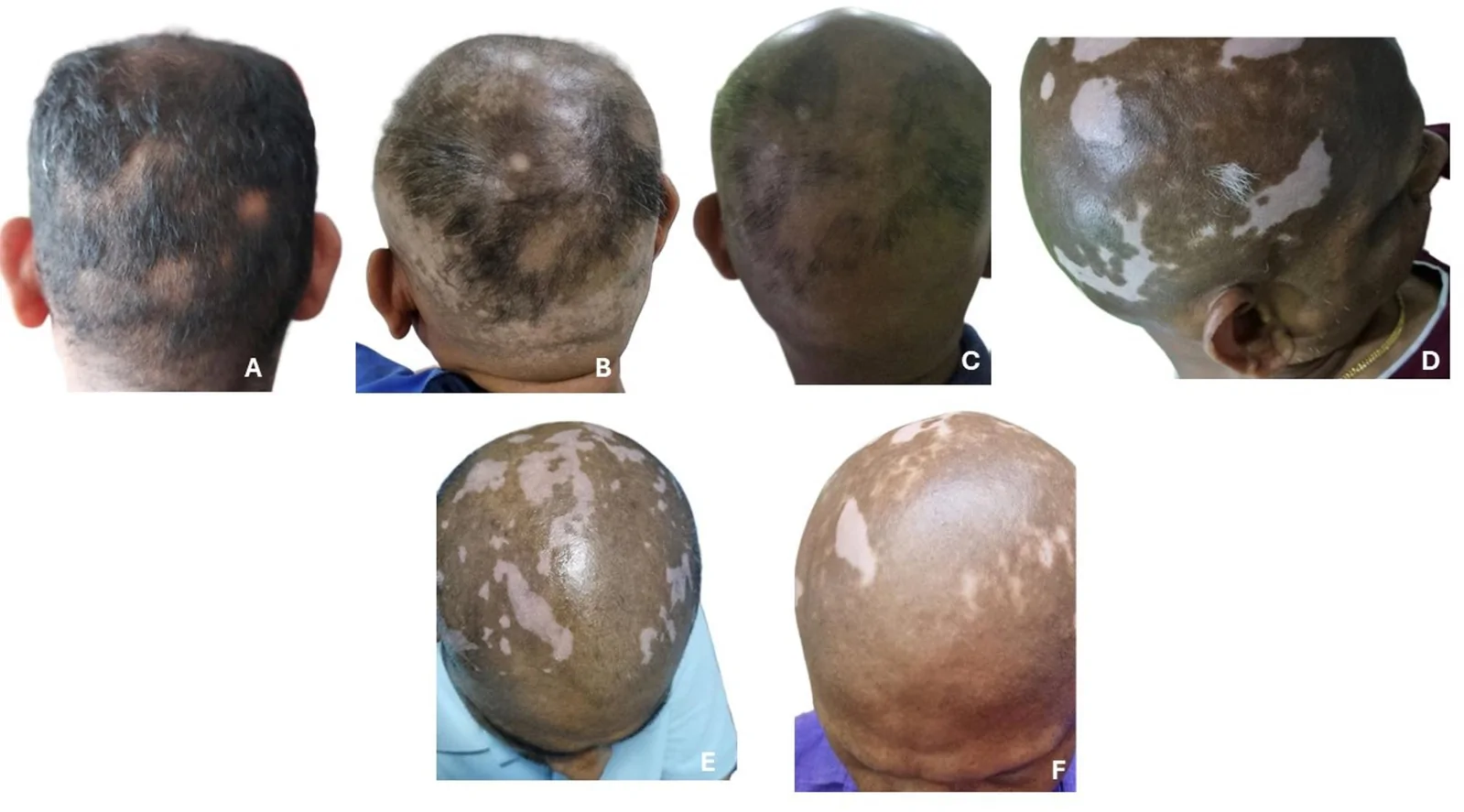
Shows the serial images (**A**-**D**) of hair loss and depigmentation following the COVID-19 vaccination. Images courtesy – Patient. Images **E** and **F** are before and after diphencyprone treatment

Review of the history indicated that ocular symptoms began 2 months after the second dose of the Pfizer–Comirnaty vaccine. Subsequently, he lost scalp hair (Fig. 6A–D), eyebrows, axillary hair, and genital hair. He had been treated by a dermatologist with diphencyprone (DPCP) for scalp vitiligo, which improved over time.(Fig. 6E, F).

On this presentation, the patient was found to have alopecia areata; pertinent blood investigations at that time are summarized in Table 1.

**Table 1:** Shows investigation done for the patient

Investigations	Value	Range
Haemoglobin	**12.5**	(14–18 g/dl)
Sex hormone binding globulin	**79**	(17–78) nmol/L
Anti-Intrinsic Factor	**71**	(< 10.0) U/mL
Anti-TPO Ab	**244**	(0–50) IU/mL
Parietal Cell Ab	**173**	(0–20) RU/mL
Cortisol 8 am	160	(185–624) nmol/L
Vitamin B12	105	(133–675) pMol/L
PTH	2.7	(0.9–6.2)pMol/ L
ACTH Plasma	50.8	(7.2–63.3) PG/ml
Total testosterone	18.8	(7.3–27.4) Nmol/L
Ferritin	**3.9**	30–400 ng/ml

TPO- Thyroid peroxidase antibody; Ab- Antibody; PTH- Parathyroid hormone ACTH- Adrenocorticotrophic hormone

The color vision screening with Ishihara was performed and was impaired in the right eye and normal in the left eye. The patient was referred to a medical retina trained inherited diseases specialist who ruled out inherited retinal diseases in lieu of gross asymmetry of presentation between the eyes. Genetic testing was not recommended as it didn’t fall into a specific pattern of inherited retinal diseases.

We diagnosed bilateral autoimmune retinopathy (AIR) in the setting of a systemic autoimmune disease, likely vaccine-related, supported by elevated anti–intrinsic factor antibody, anti–thyroid peroxidase antibody, and anti-parietal cell antibody levels, together with low cortisol, vitamin B12, and thyroid hormone levels. According to Modjtahedi et al.‘s [2] 2025 criteria from the American Academy of Ophthalmology Task Force, our patient meets probable AIR classification. He exhibited progressive asymmetric visual loss and nyctalopia-like symptoms unexplained by alternative retinal pathology, with supportive findings of abnormal ERG (reduced DA/LA responses progressing to near-flat tracings), multimodal imaging changes (OCT ellipsoid zone loss, FAF hypo-/hyperautofluorescence, FFA venous leakage), positive anti-retinal antibodies (carbonic anhydrase, aldolase, enolase, pyruvate kinase), absent intraocular inflammation, and retinal features inconsistent with inherited dystrophy.

The patient was referred to endocrinology for hypothyroxinemia and vitamin B12 deficiency and was started on levothyroxine 50 µg daily, along with iron and vitamin B12 supplementation.

Mycophenolate mofetil (MMF) was initiated at 500 mg BD after multidisciplinary discussion (ophthalmology, rheumatology, endocrinology and dermatology). Alopecia risk was negligible given that patient’s pre-existing AA which remained stable on DPCP during 14 months of follow-up.

Rheumatology was consulted pre-MMF initiation, after confirming no contraindications, MMF was commenced their input supported steroid-sparing immunosuppression given TB treatment completion and endocrine stabilization.

After 2 weeks, with normal complete blood counts and liver enzymes, the dose was increased to 1 g BD and subsequently titrated to 1.5 g BD over 14 months.

**Fig. 7:**
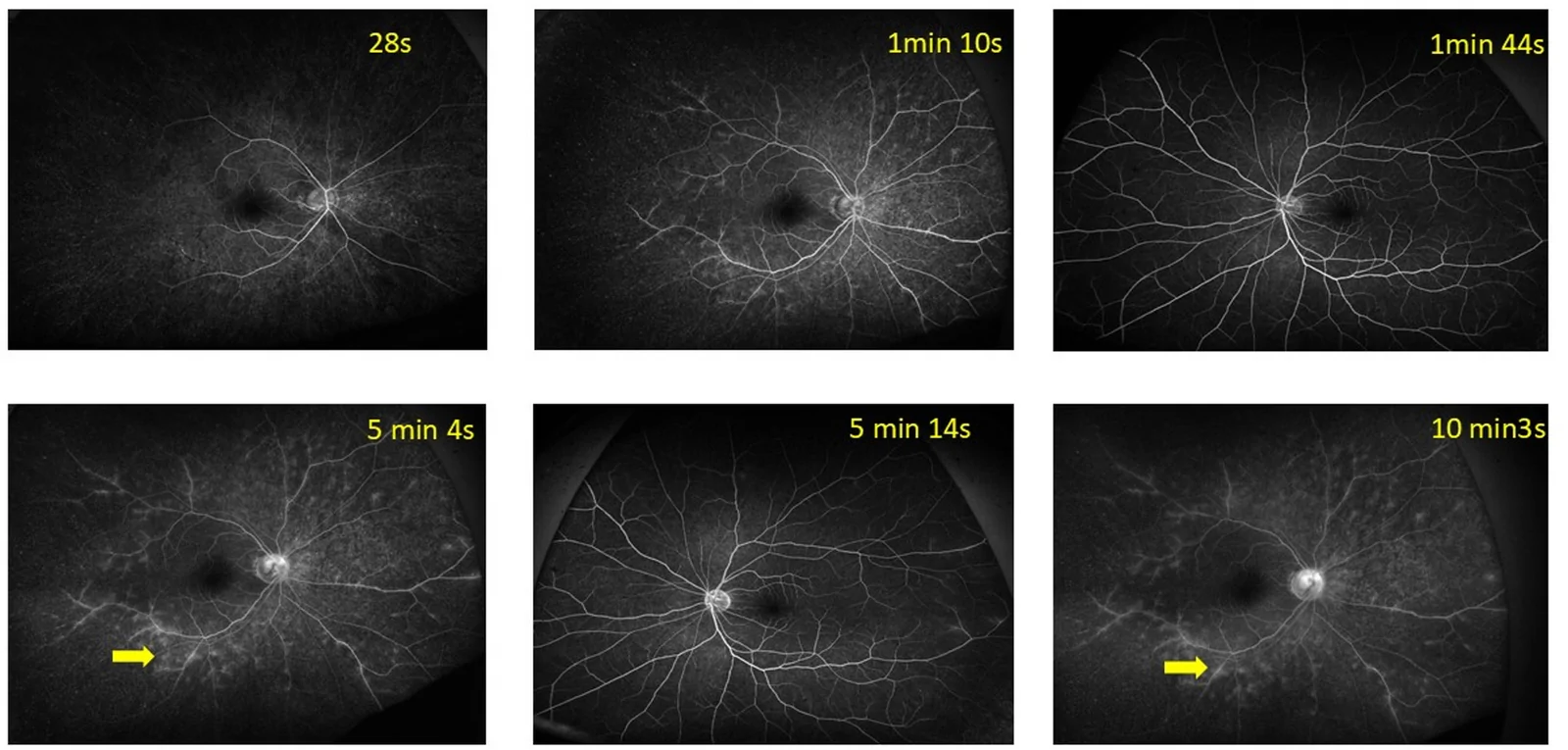
Shows Fundus fluorescein angiography in 2025, 6 months after initiation of immunosuppressive therapy showing significant reduction in the leakage in RE

At six months consult, BCVA was 20/200 in RE and 20/25 in LE. Repeat FFA demonstrated reduced vascular leakage (Fig. 7).

After intitiation of treatment, the complete absence of the EZ in the RE with thinning of the Outer Nuclear Layer (ONL) corresponds to AF signal of hypo-autofluorescence as there are no more viable photoreceptors but RPE is still present. (Fig. 2C/E and Fig. 4C/E).

Final follow up at 14 months, the patient had a stable BCVA was 20/200 in RE and 20/25 in LE with no progression of AF changes in RE. OCT changes also remained stable.

## Discussion

Vaccines against coronavirus disease 2019 (COVID-19) have been associated with ocular complications ranging from mild to severe [4–8]. Hurissi et al. reported ocular inflammation as the most common post–COVID-19 vaccination complication (47.3%), followed by optic neuritis, retinal arterial and venous occlusions, acute macular neuroretinopathy, paracentral acute middle maculopathy, and herpetic ocular infections; 42% of affected individuals had received the Pfizer–BioNTech vaccine [5]. These manifestations typically appeared within 42 days and were attributed to vaccine-induced immune responses [5]. Bolletta et al. [6]. described a broad spectrum of complications—including herpetic keratitis, scleritis, uveitis, toxoplasma retinochoroiditis, Vogt–Koyanagi–Harada (VKH) disease reactivation, retinal vasculitis, panuveitis, multiple evanescent white dot syndrome (MEWDS), nonarteritic anterior ischemic optic neuropathy (NAION), reactivation of quiescent choroidal neovascularization, and central serous chorioretinopathy (CSCR)—with a mean onset of 9.4 days after receipt of various COVID-19 vaccines [6]. Testi et al. [7] reported inflammatory events within 14 days, primarily anterior uveitis; most patients were successfully managed with topical corticosteroids, and final visual acuity was largely preserved. Scleritis and episcleritis were generally mild and rarely required intensive immunosuppression [8]. 

Our patient presented unique diagnostic challenges: asymmetric progressive vision loss initially misdiagnosed as rod–cone dystrophy despite minimal fundus changes, later progressing despite TB therapy. Unlike typical post–COVID-19 vaccine uveitis (anterior inflammation, optic neuritis, occlusions) reported within 42 days, his bilateral central hypo-AF with perimacular hyper-autofluorescence and venular leakage on FFA emerged 2 months post-Pfizer vaccination, without cells/haze—prompting AIR consideration over infectious/inflammatory aetiology.

The multisystem features further complicated attribution. Concurrent alopecia areata (stable on DPCP), Hashimoto’s thyroiditis (positive TPO/parietal cell/AIFA), and pernicious anemia (low B12/cortisol) mirrored vaccine-related MIS-V patterns [9], but onset at 2 months exceeded typical 9–14 day windows. This temporal gap, combined with positive anti-retinal antibodies and ERG progression (near-flat RE responses post-ATT), aligned with Modjtahedi criteria for probable AIR [2]. 

Therapeutically, ATT failure (BCVA drop to 20/400 RE) and steroid non-response justified immunosuppression escalation. MMF (titrated to 1.5 g BD post-rheumatology/endocrinology input) yielded partial stabilization (20/200 RE, reduced FFA leakage at 6 months)—consistent with its steroid-sparing efficacy in noninfectious uveitis/AIR. Alopecia risk was counselled given AA history, with no worsening observed.

This case underscores multimodal imaging/serology’s role in reclassifying atypical retinopathies and multidisciplinary input for vaccine-associated autoimmunity.

Modjtahedi et al. [2], on behalf of the American Academy of Ophthalmology Task Force, formulated guidelines for the diagnosis, management, and study of AIR. This diagnostic framework categorizes AIR by likelihood (probable, possible, or unlikely) to standardize disease classification [10]. Table 2 summarizes the findings in our patient that support the diagnosis of AIR. According to Modjtahedi et al.‘s [2] 2025 criteria from the American Academy of Ophthalmology Task Force, our patient meets probable AIR classification. He exhibited progressive asymmetric visual loss and nyctalopia-like symptoms unexplained by alternative retinal pathology, with supportive findings of abnormal ERG (reduced DA/LA responses progressing to near-flat tracings), multimodal imaging changes (OCT ellipsoid zone loss, FAF hypo-/hyper-autofluorescence, FFA venous leakage), positive anti-retinal antibodies (carbonic anhydrase, aldolase, enolase, pyruvate kinase), absent intraocular inflammation, and retinal features inconsistent with inherited dystrophy.

**Table 2:** Shows positive features supporting the diagnosis of AIR in our patient [2, 10]

Positive findings in our patient	Negative findings in our patient
Middle age	Nyctalopia
More acute onset of symptoms	Dyschromatopsia
No Family history of eye disease	Peripheral loss of vision
Painless photopsia	
Photoaversion	
Visual function abnormality without other apparent cause	
No “overt” intraocular inflammation (defined as less than 1 + intraocular cell or haze)	
Abnormalities on ERG (+/- visual field defects)	
Serum antiretinal antibodies	
Lack of other lesions that could explain the vision loss (including retinal lesions consistent with IRD)	

ERG- Electroretinogram

IRD- Inherited retinal disease

BCVA progression is detailed in Table 3. The RE declined from 20/80 at diagnosis to 20/400 post-ATT, improving to 20/200 by 6 months on MMF with reduced FFA leakage, and stable at 14 months on MMF 1.5 g BD; LE remained stable at 20/25 throughout.

**Table 3:** Timeline of best-corrected visual acuity (BCVA) and key treatments

Time Point	RE BCVA	LE BCVA	Treatment/Event
~ 2021 (diagnosis)	20/80	20/25	Rod–cone dystrophy label; no therapy
~ 2023 (TB suspicion)	-	-	ATT + oral steroids initiated
Post-ATT (9 months, ~ 2024)	20/400	20/25	ATT completed; progression noted
MMF start (~ late 2024)	20/400	20/25	MMF 500 mg BD; endocrinology input
2 weeks	Stable	Stable	Dose ↑ to 1 g BD (normal labs)
~ 3 months	Stable	Stable	Dose ↑ to 1.5 g BD
6 months (2025)	20/200	20/25	Reduced FFA leakage [Figure 7]
Final visit (14 months, 2026)	20/200	20/25	MMF 1.5 g BD continued, stable AF/OCT

TB- Tuberculosis; ATT- Anti Tubercular therapy; BCVA- Best corrected visual acuity; RE- Right eye; LE- Left eye; MMF- Mycophenolate mofetil; FFA-Fundus fluorescein angiography; AF- Autofluorescence; OCT- Optical coherence tomography

Thyroid peroxidase antibodies (TPO) were positive, and the patient was diagnosed with Hashimoto’s thyroiditis [11] with features of subclinical hypothyroidism. He was also positive for anti–intrinsic factor antibodies (AIFAs).

Ocular inflammation following COVID-19 vaccines could be due to molecular mimicry and bystander activation, which could postulate the reactivation of inflammation [12]. Vaccine-related multisystem inflammatory syndrome (MIS-V) has been reported in medical literature with both mRNA vaccines [Pfizer–BioNTech, Moderna] and conventional inactivated vaccines [BBIBP-CorV (Sinopharm)], with good clinical descriptions [13–16]. Although generally self-limiting, some individuals affected by MIS-V have required critical organ support in the intensive care unit (ICU).

Anti–intrinsic factor antibodies (AIFAs) are antibodies produced by the immune system that target intrinsic factor, a protein essential for the absorption of vitamin B12 in the small intestine.

Antibodies to gastric parietal cells are associated with autoimmune gastritis and pernicious anemia. The parietal cells of the stomach secrete intrinsic factor, which is necessary for the absorption of vitamin B12, essential for erythropoiesis.

Topical immunotherapy with DPCP is widely used in patients with alopecia areata (AA). It can produce contact dermatitis that is believed to decrease the Th1 response, which is predominant in alopecia areata [17]. While alopecia is an uncommon side effect of MMF (reported in 3–20% of cases across transplant/lupus/uveitis trials, often mild/reversible) [18, 19], our patient was not affected as he did not have any hair in the body and DCP was improving only the hypopigmented patches and did not result in new hair growth.

## Conclusion

We report a case of probable autoimmune retinopathy as part of a multisystem autoimmune syndrome—including alopecia areata, Hashimoto’s thyroiditis, and pernicious anemia—that arose 2 months after COVID-19 vaccination in a previously healthy man. Through persistent multimodal imaging, serial ERG monitoring, and targeted serology, we unravelled an initial misdiagnosis of rod–cone dystrophy and TB vasculitis. Mycophenolate mofetil brought partial visual stabilization after failed ATT, underscoring the value of multidisciplinary collaboration in navigating these rare, challenging post-vaccine presentations.

## Data Availability

The data generated and/or analysed during the current study are available from the corresponding author on reasonable request.

## Abbreviations

AIR: Autoimmune retinopathy
BCVA: Best-corrected visual acuity
RE: Right eye
LE: Left eye
OCT: Optical coherence tomography
FFA: Fundus fluorescein angiography
TB: Tuberculosis (from T-spot TB)
AF: Autofluorescence
ERG: Electroretinography / Electroretinogram
ARA: Antiretinal autoantibodies
DA: Dark adaptation
LA: Light adaptation
PERG: Pattern ERG
ATT: Anti-tubercular treatment
PET: Positron emission tomography
DPCP: Diphencyprone
BD: Twice daily
COVID-19: Corona virus disease 19
TPO: Thyroid peroxidase (antibody)
AIFA: Anti-intrinsic factor antibody
MIS-V: Multisystem inflammatory syndrome in vaccine
AA: Alopecia areata
Ab: Antibody
PTH: Parathyroid hormone
ACTH: Adrenocorticotrophic hormone
IRD: Inherited retinal disease
MEWDS: Multiple evanescent white dot syndrome
NAION: Non-arteritic anterior ischemic optic neuropathy
CNV: Choroidal neovascularization
CSCR: Central serous chorioretinopathy
VKH: Vogt-Koyanagi-Harada
ICU: Intensive care unit
